# Cerebral oximetry monitoring versus usual care for extremely preterm infants: a study protocol for the 2-year follow-up of the SafeBoosC-III randomised clinical trial

**DOI:** 10.1186/s13063-023-07653-x

**Published:** 2023-10-07

**Authors:** Marie Isabel Rasmussen, Mathias Lühr Hansen, Adelina Pellicer, Christian Gluud, Eugene Dempsey, Jonathan Mintzer, Simon Hyttel-Sørensen, Anne Marie Heuchan, Cornelia Hagmann, Ebru Ergenekon, Gabriel Dimitriou, Gerhard Pichler, Gunnar Naulaers, Guoqiang Cheng, Jakub Tkaczyk, Hans Fuchs, Monica Fumagalli, Saudamini Nesargi, Siv Fredly, Tomasz Szczapa, Anne Mette Plomgaard, Bo Mølholm Hansen, Janus Christian Jakobsen, Gorm Greisen

**Affiliations:** 1 Department of Neonatology, Copenhagen University Hospital - Rigshospitalet, Blegdamsvej 9, Copenhagen Ø, 2100 Denmark; 2 Centre for Clinical Intervention Research, Copenhagen Trial Unit, The Capital Region, Copenhagen University Hospital - Rigshospitalet, Copenhagen, Denmark; 3 Department of Neonatology, La Paz University Hospital, Madrid, Spain; 4 Department of Regional Health Research, The Faculty of Health Sciences, University of Southern Denmark, Odense, Denmark; 5 Infant Research Centre and Department of Paediatrics and Child Health, University College Cork, Cork, Ireland; 6 Department of Pediatrics, Division of Newborn Medicine, Mountainside Medical Center, Montclair, NJ USA; 7 Department of Intensive Care, Copenhagen University Hospital – Rigshospitalet, Copenhagen, Denmark; 8 Department of Neonatology, Royal Hospital for Children, Glasgow, UK; 9 Department of Neonatology, Children’s University Hospital of Zürich, Zurich, Switzerland; 10 Department of Neonatology, Gazi University Hospital, Yenimahalle, Ankara, Turkey; 11 Department of Pediatrics, NICU, University General Hospital of Patras, Patras, Greece; 12 Department of Pediatrics, Medical University of Graz, Graz, Austria; 13 Department of Neonatology, University Hospital Leuven, Louvain, Belgium; 14 Department of Neonatology, Children’s Hospital of Fudan University, Shanghai, China; 15 Department of Neonatology, University Hospital Motol, Prague, Czech Republic; 16 Division of Neonatology and Pediatric Intensive Care Medicine, Center for Pediatrics and Adolescents Medicine, Medical Center, University of Freiburg, Freiburg, Germany; 17 Fondazione IRCCS Ca’ Granda Ospedale Maggiore Policlinico Milan, Milan, Italy; 18 Department of Clinical Sciences and Community Health, University of Milan, Milan, Italy; 19 St. Johns Medical College Hospital, Bengaluru, India; 20 Department of Neonatology, Oslo University Hospital, Oslo, Norway; 21 II Department of Neonatology, Poznan University of Medical Sciences, Poznań, Poland; 22 Department of Pediatrics, Copenhagen University Hospital, Hvidovre, Denmark; 23 Department of Paediatrics and Adolescent Medicine, Copenhagen University Hospital, Hilleroed, Denmark

**Keywords:** Randomised clinical trial, Preterm, Near-infrared spectroscopy, Protocol, Follow-up, Neurodevelopment, Brain injury

## Abstract

**Background:**

In the SafeBoosC-III trial, treatment guided by cerebral oximetry monitoring for the first 72 hours after birth did not reduce the incidence of death or severe brain injury in extremely preterm infants at 36 weeks’ postmenstrual age, as compared with usual care. Despite an association between severe brain injury diagnosed in the neonatal period and later neurodevelopmental disability, this relationship is not always strong. The objective of the SafeBoosC-III follow-up study is to assess mortality, neurodevelopmental disability, or any harm in trial participants at 2 years of corrected age. One important challenge is the lack of funding for local costs for a trial-specific assessment.

**Methods:**

Of the 1601 infants randomised in the SafeBoosC-III trial, 1276 infants were alive at 36 weeks’ postmenstrual age and will potentially be available for the 2-year follow-up. Inclusion criteria will be enrollment in a neonatal intensive care unit taking part in the follow-up study and parental consent if required by local regulations. We aim to collect data from routine follow-up programmes between the ages of 18 and 30 months of corrected age. If no routine follow-up has been conducted, we will collect informal assessments from other health care records from the age of at least 12 months. A local co-investigator blinded to group allocation will classify outcomes based on these records. We will supplement this with parental questionnaires including the Parent Report of Children’s Abilities—Revised. There will be two co-primary outcomes: the composite of death or moderate or severe neurodevelopmental disability and mean Bayley-III/IV cognitive score. We will use a 3-tier model for prioritisation, based on the quality of data. This approach has been chosen to minimise loss to follow-up assuming that little data is better than no data at all.

**Discussion:**

Follow-up at the age of 2 years is important for intervention trials in the newborn period as only time can show real benefits and harms later in childhood. To decrease the risk of generalisation and data-driven biased conclusions, we present a detailed description of the methodology for the SafeBoosC-III follow-up study. As funding is limited, a pragmatic approach is necessary.

**Trial registration:**

ClinicalTrials.gov NCT05134116. Registered on 24 November 2021.

**Supplementary Information:**

The online version contains supplementary material available at 10.1186/s13063-023-07653-x.

## Background

Worldwide, approximately 15 million infants are born preterm (below 37 weeks’ gestational age) each year [1]. Hereof, around 50,000 are born extremely preterm (below 28 weeks’ gestational age) in countries where neonatal intensive care is offered routinely [2]. Currently, mortality is around 20%, but those surviving have a high risk of neonatal brain injury and subsequently long-term neurodevelopment disability [3]. The risk of brain injury is especially high during the first days of life, as the infant transitions from intra- to extrauterine life. The combination of immature organs and impaired cerebral autoregulation may cause large fluctuations in cerebral blood flow [4], detectable by cerebral oximetry using near-infrared spectroscopy (NIRS) monitoring. These fluctuations in the immature brain may lead to cerebral hypoxia and hyperoxia and can cause brain injury [5]. Brain injury acquired in the neonatal period may result in neurodevelopmental disability later in life. As many as 40% of surviving extremely preterm infants are diagnosed with neurodevelopmental disability such as cerebral palsy, cognitive and neurosensory deficits, attention-deficit disorder, and/or major psychiatric disorders before school age [6]. These early disabilities usually result in life-long consequences for the children and their families, such as reduced quality of life as well as increased health care and educational costs [7, 8]. Evidence suggests that cerebral hypoxia is associated with the risk of brain injury as well as death in extremely preterm infants [9].

### The SafeBoosC-II trial

The SafeBoosC phase-II trial demonstrated that cerebral oximetry monitoring in combination with a treatment guideline reduced the burden of cerebral hypoxia and hyperoxia by more than 50%, compared to blinded cerebral oximetry monitoring, during the first 72 h after birth [10]. Furthermore, there were trends towards reduced mortality and occurrence of severe brain injury assessed at 36 weeks’ postmenstrual age [10]. In total, 115/135 (85%) participants alive at 24 months’ corrected age were followed up to test if the intervention was beneficial in terms of improving neurodevelopment later in life. No differences were found between the unblinded cerebral oximetry group and blinded cerebral oximetry group regarding the mean mental developmental index assessed by the Bayley II (89.6 ± 19.5 versus 88.4 ± 14.7, *p* = 0.77) or the total Ages and Stages Questionnaire score (215 ± 58 versus 213 ± 58, *p* = 0.88) [11]. The number of participants with moderate or severe neurodevelopmental disability was also similar, with ten (15%) in the cerebral oximetry group and six (12%) in the blinded cerebral oximetry group (*p* = 0.58) [11]. However, the SafeBoosC-II trial was not powered to detect a relevant difference in any of the long-term clinical outcomes and thus there is a possibility that the neutral results were due to type II errors. Based on the above, the larger SafeBoosC-III trial has been conducted, to test the effect of the intervention on clinical outcomes.

### The SafeBoosC-III trial

SafeBoosC-III is a multi-centre, international, pragmatic phase III clinical trial investigating the effects of treatment guided by cerebral oximetry monitoring in extremely preterm infants [12]. The hypothesis was that treatment guided by cerebral oximetry monitoring in the first 72 h after birth in extremely preterm infants would result in a reduction of death or severe brain injury at 36 weeks’ post-menstrual age, as compared with usual care. Infants randomised to the cerebral oximetry group were monitored during the first 72 h after birth and received cardio-respiratory support guided by cerebral oximetry monitoring [13]. The cerebral oximetry sensor was placed on the head within 6 h of birth. The usual care group was treated according to local clinical guidelines without cerebral oximetry. A total of 1601 infants were randomised across 70 sites from Europe, the USA, China, and India and 1579 (98.6%) were evaluated for the primary outcome. At 36 weeks’ post-menstrual age, death or severe brain injury occurred in 272 of 772 participants (35.2%) in the cerebral oximetry group as compared with 274 of 807 participants (34.0%) in the usual care group (RR with cerebral oximetry, 1.03; 95% CI 0.90 to 1.18; *P* = 0.64) [14]. The trial is registered at clinicaltrials.gov (NCT03770741).

The original sample size of the SafeBoosC-III trial was calculated based on the primary outcome, which is a composite of death or severe brain injury at 36 weeks’ post-menstrual age [15]. Severe brain injury was defined as intraventricular haemorrhage grade III or IV, cystic periventricular leukomalacia, cerebellar haemorrhage, post-haemorrhagic ventricular dilatation or cerebral atrophy detected on any of the routine cerebral ultrasound scans that were routinely performed in the trial participants up until 36 weeks’ post-menstrual age or discharge to home, whichever came first [16].

Assessment at 2 years’ corrected age is commonly used clinically as well as for research purposes [17]. The predictive value of such assessments for later function is relatively poor for the individual child. However, on a group level, the assessments demonstrate the expected differences between extremely preterm-born children and children born at term. Therefore, the assessments are likely to be valuable as outcome measures in randomised clinical trials. With the SafeBoosC-III trial having randomised 1601 participants, there is a potential to achieve sufficient power for a meaningful assessment of the intervention’s effect on long-term neurodevelopment.

## Methods

**Fig. 1:**
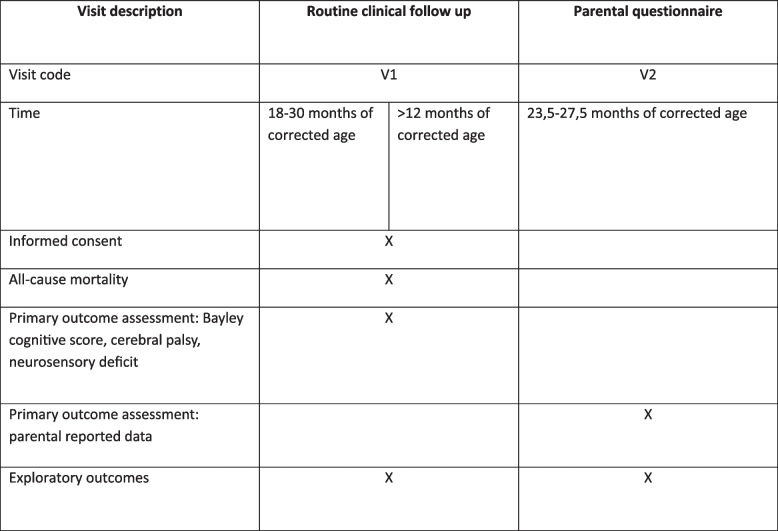
SPIRIT schedule of enrollment and assessment

The study has been designed according to the SPIRIT guidelines (Fig. 1 and Appendix A) [18].

The objective of the SafeBoosC-III follow-up study is to investigate the benefits and harms of treatment guided by cerebral oximetry monitoring in extremely preterm infants during the first 72 hours after birth, assessed at 2 years’ corrected age, as compared with usual care.

The hypothesis is that the intervention will decrease a composite of death or moderate or severe neurodevelopmental disability at 2 years’ corrected age, and/or increase cognitive function in survivors assessed by the Bayley-III/IV test, with insignificant harms.

### Inclusion criteria

Participation in the SafeBoosC-III trial, enrolment in a site taking part in the SafeBoosC-III follow-up study, and parental consent if required by local regulations.

### Primary outcomes

The two co-primary outcomes are:

1. Death or moderate or severe neurodevelopmental disability (NDD)

A child will be classified with moderate or severe NDD if any of the four following conditions are present:

- Cerebral palsy with functional impairment corresponding to Gross Motor Function Classification Score (GMFCS) ≥ 2
- A score below − 2 standard deviations from the norm of a standardised developmental assessment (if using the Bayley-III/IV test, the cognitive score cut-off will be < 85), or an informal classification of moderate or severe NDD
- Vision impairment defined as moderately reduced vision, or only being able to perceive light or light-reflecting objects, or blind in one eye with good vision in the contralateral eye
- Hearing impairment defined as hearing loss corrected with aids (usually moderate 40 to 70dBHL) or some hearing but loss not corrected by aids (usually severe 70 to 90dBHL).

2) Bayley-III/IV mean cognitive score

### Exploratory outcomes

The exploratory outcomes are as follows:

- Daily medication for the last 2 months (yes/no)
- Any other chronic illness (defined as any problem which has been diagnosed by a doctor and which is expected to last more than a few months, causes problems in everyday life, or is a risk of early death or disability)
- Mean head circumference
- Mean height
- Mean body weight, with one decimal

All components of the co-primary outcomes will be reported for the two groups separately, including effect estimates with confidence intervals, and will be taken into consideration when interpreting the results.

### Outcome assessment tools

Routine follow-up varies greatly from site to site and we aim to collect data on as many children as possible. The following outcome assessment tools may be used to collect data on children in the SafeBoosC-III FU. We defined three tiers of data: (1) routine clinical data from healthcare records, (2) parental questionnaire, and (3) informal assessments of neurodevelopment. The ‘3-tier model’ also represents a principle for prioritisation of the data source for classifying the co-primary dichotomous outcome death or moderate or severe NDD.

#### Tier 1: routine clinical data

##### Medical examination

A medical examination will ideally include measurement of head circumference; height and weight and the child’s vision and hearing will be tested either formally or informally. Signs of cerebral palsy will be documented and a motor ability scale will be used [19]. Finally, the presence of chronic illnesses and/or medication usage will be documented. However, as described previously, routine follow-up varies from site to site and only diagnoses, for example, a diagnosis of cerebral palsy, are to be recorded in the electronic case report form (eCRF).

##### Neurodevelopmental assessments and tests

Some sites do formal assessments of neurodevelopment as a part of their routine follow-up. Most sites in the SafeBoosC-III FU use the Bayley Scales of Infant Development (Bayley) assessment and therefore it has been chosen for the quantitative co-primary outcome. The Bayley is a commonly used neurodevelopmental assessment tool to identify neurodevelopment delays in children from 1 to 42 months of age [20].

As of today, the most used neurodevelopmental assessment tool is the Bayley-III. Some sites may switch to the Bayley-IV during the SafeBoosC-III FU study. In published data from the provider of the Bayley assessments, it is argued that there is evidence to support the accuracy and validity of the Bayley-IV scores [21]. Furthermore, as the Bayley-IV (2019) retains the same subcategories and assessment methods as Bayley-III, it is predicted to yield similar scores to the Bayley-III. No randomised clinical trials have used and published results using the Bayley-IV as an outcome measure yet, and therefore, the scores will be treated as equivalent to the Bayley-III scores in the co-primary outcome Bayley-III mean cognitive score. In the event of a possible difference between the average Bayley-III and IV scores, the nature of a randomised trial and block randomisation will equally distribute the differences between the cerebral oximetry and usual care group. Furthermore, we will conduct a sensitivity analysis investigating a possible influence on treatment effect caused by differences in Bayley-III and IV scores.

For the co-primary outcome Bayley-III/IV mean cognitive score, the unadjusted mean scores from the cerebral oximetry and usual care group will be compared. If the Bayley-III/IV cognitive score is used to evaluate cognitive function for the co-primary outcome death or moderate or severe NDD, the cognitive function cut-off will be < 85, as done in previous trials and recommended by experts [22].

Data from other formal neurodevelopmental assessments such as the Griffiths, Denver Developmental Screening Test, and Ages and Stages Questionnaire will be collected only to be used for the co-primary outcome death or moderate or severe NDD, in case a Bayley has not been performed. If more than one test has been performed, they will be prioritised in this order: Bayley IV/III, Bayley II, Griffiths III, Gessell, ASQ, Denver Developmental Screening, Peabody, and any other.

#### Tier 2: parental questionnaire

The parental questionnaires will act as a common denominator in what may be a heterogeneous clinical data set. The parental questionnaire will be comprised of the Parent Report of Children’s Abilities – Revised (PARCA-R) non-verbal cognitive scale and a health and development questionnaire.

##### PARCA-R

PARCA-R is a parental questionnaire that can be used to assess children’s non-verbal cognitive and language development at 24 months’ corrected age. The PARCA-R provides high test–retest reliability and correlates well with the cognitive score of the Bayley-III [23–25]. The questionnaire has been used as a 2-year outcome measure in multiple clinical trials [26] and has furthermore been translated into several languages. For this study, an online platform will be developed, where parents of the participating children will be invited to complete the non-verbal cognitive part of the PARCA-R questionnaire, in their respective languages. This typically takes less than 10 min to complete [27]. The full PARCA-R will not be used, since a validated language component is not available in all countries; PARCA-R has only been standardised for use in the UK. The PARCA-R must be assessed on children between 23.5 and 27.5 months’ corrected age. Children will be classified as having moderate or severe NDI when scores correspond to a scale score of − 2 standard deviations from the norm [28].

If a neurodevelopmental assessment (i.e. Bayley, Griffiths) of the child is not available, PARCA-R will be used for classification for the co-primary outcome death or moderate or severe NDD. When more than four items are missing, the scores will not be calculated, but will be categorised as ‘unknown’.

##### Health and development questionnaire

The PARCA-R questionnaire will be supplemented with a number of health and developmental questions to be answered by the parents as well. The questions focus mainly on the individual components of the moderate or severe NDD outcome definition, as well as quality of life questions. It will be available on the online platform as well. The coding of the health and development questionnaire for the co-primary outcome moderate or severe NDD can be found in Appendix B.

#### Tier 3: informal assessments of neurodevelopment

If no formal assessments of the child have been conducted between the ages of 18 and 30 months’ corrected age, the blinded assessor will provide an informal assessment of the presence or absence of moderate or severe NDD based on all available information on the child from at least 12 months’ corrected age. If no formal neurodevelopmental assessment (i.e. Bayley, Griffiths) of the child has been conducted, the local co-investigator will also provide an informal assessment of moderate or severe NDD.

For additional details on the 3-tier model, see Appendix C.

#### Power estimations for the co-primary outcomes

The sample size calculation of 1600 participants for the SafeBoosC-III trial was based on mortality and the prevalence of survivors with severe brain injury in the SafeBoosC-II trial, i.e. a 22% relative risk reduction in the composite primary outcome from 34 to 26.5% (absolute risk reduction of 7.5%), at an alpha level of 5%, and a power of 90% [15]. Of the 1601 participants randomised, 1276 were alive at 36 weeks’ post-menstrual age and will potentially be available for the SafeBoosC-III follow-up study. The following calculations were carried out using the software OpenEpi (Dean AG, Sullivan KM, Soe MM. OpenEpi: Open Source Epidemiologic Statistics for Public Health, Version.)

#### Death or moderate or severe neurodevelopmental disability

Based on answers to a questionnaire on systematic routine follow-up from participating sites, as well as implementing parental questionnaires and informal assessments to classify neurodevelopment, we believe it is reasonable to expect a minimal loss to follow-up and expect the total sample size for the outcome death or moderate or severe NDD to near 1600 participants.

Based on results from two randomised clinical trials investigating neuroprotection in preterm infants [29, 30], it is estimated that the proportion of children with the outcome death or moderate or severe NDD will be 50% in the usual care group. An indicative power calculation shows that if we want to test an absolute risk difference of 8%, between the experimental and control group, at an alfa of 2.5% and a sample size of 800 participants in each group, i.e. a total of 1600, we will reach a power of 80% for this outcome.

#### Bayley-III/IV mean cognitive score

Based on answers to a questionnaire on systematic routine follow-up, it is expected that 65% of the sites will be able to provide data from a Bayley-III/IV assessment around 2 years’ corrected age for each child. Assuming that these sites enrol their proportion of the eligible participants to the SafeBoosC-III trial, approximately 850 participants will be available for Bayley-III/IV assessments at 2 years of age.

An indicative power estimation shows that if we want to test a mean difference of five points on the Bayley cognitive score, with a standard deviation of 20 (Cohens *d*’ 0.25) between the cerebral oximetry and the usual care group, at a 2.5% alfa-level, with a sample size of 425 participants from each group in the follow-up study, i.e. a total of 850, we will reach a power of 90%.

#### Primary analyses

The primary analyses of all outcomes will be based on the intention-to-treat population. Mixed-effect linear regression and mixed-effect logistic regression will be used to analyse the dichotomous and continuous co-primary outcomes, respectively. In the regression models, ‘site’ will be included as a random effect, while ‘gestational age below or above 26 weeks of postmenstrual age’ and ‘group allocation’ will be included as fixed effects. To correct for multiple testing, the threshold for statistical significance will undergo Bonferroni adjustment, and thus a *p*-value of 0.025 for each primary outcome is chosen. Superiority of the intervention will only be claimed if at least one of the two co-primary outcomes is statistically significant at this level. All other outcome results will be considered hypothesis-generating. In addition, we will perform several pre-defined sensitivity analyses to inform the interpretation of the results of the primary analysis. A full statistical analysis plan will be developed and submitted for publication before the analysis of the SafeBoosC-III FU data.

#### Data extraction and coding

All data extraction from health care records and data entry into the e-CRF will be conducted by an assessor who is blinded to group allocation. The co-primary outcome Bayley-III/IV mean cognitive score will be extracted from the health care records of the Bayley-III/IV test. The co-primary outcome death or moderate or severe NDD will primarily be based on an assessment of available health care records, from when the child was between 18 and 30 months’ corrected age. If more than one formal neurodevelopmental assessment has been conducted, the score of the latest should be used. If formal assessment of all four components is not available, the missing components will be substituted with answers from parental questionnaires (PARCA-R and health and development questionnaire, if available) as a step in the final data analysis, if these are available. The data collection and management for this study will utilise the OpenClinica open source software, version 3.1. Copyright OpenClinica LLC and collaborators, Waltham, MA, USA, www.OpenClinica.com hosted by the Copenhagen Trial Unit. The answers from the questionnaires will be reported web-based into REDCap hosted at The Capital Region of Denmark by the parents or by the investigator who provides the questionnaire in the follow-up clinic or via phone.

#### Blinding

Due to the nature of the trial intervention, children and their parents were not blinded to treatment allocation, and therefore, completion of parental reported questionnaires cannot be blinded. However, a co-investigator who is blinded to group allocation will look through all relevant health care records, do classifications, and report data into the eCRF. To ensure that the co-investigator who will do the outcome assessment is blinded, the principal investigator and co-investigator from each site must develop a local blinding procedure describing the workflow. This process will be approved by the central trial unit before the study commencement. Thus, all outcome data except for the parental-reported questionnaires are assessed blindedly. Data managers, statisticians, and those writing the abstracts as well as drawing conclusions will also be blinded.

#### Ethical review

The need for supplementary approval of the follow-up study by a Research Ethics Committee will differ among countries. In some countries, no REC approval may be necessary. In some countries, a Research Ethics Committee approval may be an addition to the approval of the randomised clinical trial. As the SafeBoosC-III follow-up study does not include exposures to any additional interventions, there are no safety risks for children. Thus, an interim analysis is not necessary.

#### Consent

When infants were randomised in the SafeBoosC-III trial, the parents were made aware of the possibility of a potential follow-up study and permission to contact families as foreseen in the information sheet the parents were given at inclusion. The need for explicit consent for using clinical data from health care records for the SafeBoosC-III FU may differ between countries. In some countries, turning up for examination or answering questionnaires will be considered sufficient, implicit consent. Some centres will send out consent forms by mail while some will collect consent when the families come to the routine follow-up appointments. We have supplied the participating centres with a standard consent template, which may be altered according to local needs.

#### Data management plan

All the participants data are protected in accordance with the Danish Act on the processing of personal data and the Danish Health Act. Data will be stored in accordance with guidelines issued by the Danish Data Protection Agency, where the follow-up study will also seek approval from. Data are pseudonymised, and only site numbers and study numbers are used to identify children. Personal identifying information linking to study numbers will be kept in the trial master file at the local site. A central monitoring plan has been developed to maximise data quality. This will be done by identifying centres with missing data. This is possible since the date of birth of the participants of the randomised trial is known. Furthermore, centres with predefined quality deficiencies or noteworthy data deviations as identified by visual inspection of data distributions. Primary investigators of these centres will be contacted to remedy deficiencies, if possible. This procedure will be re-iterated every third month. The central data monitoring plan is available in the Supplementary Appendix on page 13.

Six months after the acceptance of the main publication presenting the primary outcome of the study, the dataset will be transferred to the Danish data archive, after re-coding of variables (birth weight, gestational age, sex, site, study number) that may be used for reidentification. This data set can be made available for others as decided by the trial steering committee. Due to the residual risk of re-identification, the dataset will not be placed in public space.

#### Publication plan

Once all data from the study has been analysed, the results will be published in a peer-reviewed international journal. Members of the steering group will be offered authorship. Furthermore, one investigator per participating site can obtain co-authorship. All authors must fulfil the ‘Vancouver Criteria’. The blinded assessor completing the data entries will obtain non-byline co-authorship. Ancillary studies with results potentially affecting the main study or compromise its publication shall not be published before the main results of the study have been published, as decided by the trial steering committee.

#### Timeline

The first randomised infant in SafeBoosC III reached 2 years of corrected age in September 2021, while the last follow-up assessment is expected to be completed around autumn of 2024, which is when the last infant randomised will reach 2 years of corrected age plus 6 months of the reserve.

## Discussion

The SafeBoosC-III trial tested if treatment guided by cerebral oximetry during the first 72 hours after birth could decrease the risk of death or severe brain injury at 36 weeks’ post-menstrual age in extremely preterm infants. No significant difference was found between the cerebral oximetry and the usual care group [14]. The SafeBoosC-III follow-up study will collect data on trial participants at 2 years’ corrected age to assess mortality and neurodevelopmental disability. Most importantly, this will allow for an assessment of long-term harms.

### Motivation: brain injury in the neonatal period and later neurodevelopment

Neurodevelopmental disability is the brain-specific patient-relevant outcome and neonatal brain injury is not a perfect surrogate outcome. Despite an association between severe brain injury diagnosed in the neonatal period and later neurodevelopmental disability, this relationship is not always strong. Not all infants with brain injury on ultrasound scans suffer from later neurodevelopment complications [31]. On the other hand, among extremely preterm infants with no ultrasound abnormalities, 23% had delayed mental development and 26% had delayed psychomotor development at 2 years of age [32].

One of the most common therapies given in the neonatal intensive care unit is oxygen. Oxygen is also an intervention of the SafeBoosC-III treatment guideline, which recommends adjustments of cardio-respiratory support to keep cerebral oxygenation above the hypoxic threshold. It has been shown that certain brain structures, such as white matter, are especially vulnerable to hypoxia [9]. On the other hand, with hypoxia a higher need for oxygen occurs which is also associated with white matter abnormalities and adverse neurodevelopment [33]. White matter injuries are difficult to detect on cerebral ultrasound, and thus, the results of early hypoxia and subsequent white matter injury may not be seen until later in childhood.

Oxygen therapy and possibly toxicity are also a threat to other organs. Premature infants are more sensitive to oxidative stress and have decreased protective mechanisms compared to infants born at term [34]. Hyperoxia, most likely to be induced by oxygen therapy, is well known to contribute to the development of bronchopulmonary dysplasia as well as retinopathy of prematurity. However, the extent of these diseases may be difficult to assess till after 36 weeks’ post-menstrual age. Of long-term effects, moderate to severe bronchopulmonary dysplasia contributes to a poorer resistance to respiratory viral infections in adulthood as well as the risk of neurodevelopmental disabilities. Oxygen-induced retinopathy of prematurity is well known to contribute to blindness [35].

To our knowledge, only one previous study has investigated the long-term outcome of cerebral oximetry monitoring during the first 72 h after birth. This was evaluated in the SafeBoosC-II trial where 115 participants were followed up and showed no differences between the intervention groups [11]. There are multiple challenges in conducting the SafeBoosC-III follow-up study.

#### Challenge 1: not planned initially

The SafeBoosC-III FU was described as an ‘ancillary’ study in an appendix in the SafeBoosC-III trial protocol [12]. Due to uncertainties about funding and the strength of the collaboration among the participating sites across many countries, no plans or agreements were made initially. As the participation, support of the trial and randomisations increased, it was decided by the steering committee that a follow-up study should be conducted. Like the SafeBoosC-III trial, the SafeBoosC-III FU is a pragmatic, low-budget, investigator-initiated study and the trial sponsor is not able to support local sites financially. Therefore, we refrained from a trial-specific follow-up with standardised examination methods. Instead, we will rely on chart review of routinely collected clinical data supplemented by parental questionnaires. However, routine follow-up of preterm infants is done differently in participating sites. Some sites are required by law to follow up on extremely preterm infants while others do not have a follow-up clinic at all. Thus, plans for participant retention and completion of follow-up will depend on the participating centres’ routines for follow-up of extremely preterm.

Solution: After a survey among the participating sites, data collection was planned to minimise the work for investigators, reduce the risk of ‘study-fatigue’, and optimise the compatibility of the reported data. We developed a 3-tier model that prioritises the data used to classify moderate or severe NDD. For a description of the 3 tiers, please see the ‘Methods’ section. We have chosen this strategy to minimise the number of participants lost to follow-up, since we consider that even a casual clinical note on a small child with an acute condition needing health care may provide a better estimate of the presence or absence of moderate or severe neurodevelopmental disability, than does imputing from the data collected in the SafeBoosC-III until 36 weeks’ post-menstrual age. The robustness of this strategy will be tested by statistical sensitivity analyses. This 3-tier model may also be relevant for other follow-up studies which do not have the funding to pay for trial-specific tests or examinations.

#### Challenge 2: loss to follow-up

Loss to follow-up is a major challenge for neonatal trials. Loss to follow-up varies from 25 to 50% in most trials [36]. Patients who are lost to follow-up tend to differ from those who remain in the study, with the most important factor being socioeconomic status [37]. Loss to follow-up is also more common in children with neurodevelopmental disability [38]. Both impose a risk of attrition bias. In our study, it is unlikely that loss to follow-up will be biased in terms of the group allocation since the intervention was only an initial part of a long clinical course.

Solution: To reduce the bias induced by differential loss to follow-up, sites that do not expect to provide good follow-up rates will be excluded and children will only be accounted for in sites which have chosen to actively participate.

#### Challenge 3: statistical power

SafeBoosC-III was powered to detect a reduction in death or severe brain injury from 34 to 26% which corresponds to a 22% relative risk reduction [15]. Since mortality after 36 weeks’ post-menstrual age is unlikely to be influenced by the intervention, and factors other than brain injury contribute to neurodevelopmental disability, the effect of the intervention is expected to be less at 2 years of age. Therefore, the power of the SafeBoosC-III follow-up study is less to show a difference on the dichotomous co-primary outcome death or moderate or severe NDD at 2 years’ corrected age.

Solution: We will have two co-primary outcomes. By this, we combine a chance to detect a benefit in a directly patient-relevant outcome, i.e. neurodevelopmental disability, with a high likelihood of being able to detect a benefit in terms of higher cognitive scores, even though it is needed to adjust the *p*-value for two outcomes. Although early childhood cognitive scores are not directly patient-relevant, they are correlated with later IQ, school performance, and educational achievements [6].

Due to the expected non-participation of some sites and the loss to follow-up in those sites who do take part, the power will be somewhat reduced. At 30% fewer participants, the power for the dichotomous outcome would be reduced from 80 to 70%. This will be considered when interpreting our results.

#### Challenge 4: blinding

The blinding of the clinical assessments behind the health care records cannot be controlled. It is, however, unlikely that the group allocation is in focus at the time of follow-up after discharge. The intervention was delivered during the first 72 h after birth of a hospital admission lasting 2–4 months.

Solutions: To reduce potential bias, each site must develop a procedure whereby the investigator can read all relevant health care records, do the necessary classifications, and report into a fully structured web-based eCRF. The eCRF will perform the 3-tier classification for the co-primary outcome moderate or severe neurodevelopmental disability, limiting possible bias and random error.

#### Challenge 5: assessment of cognitive scores

Routine follow-up assessments are done differently across sites participating in the SafeBoosC-III FU. Formal assessments of neurodevelopment are commonly the Bayley II, III, and IV as well as the Griffiths or Denver Developmental Screening and others. Some sites may routinely use questionnaires such as the PARCA-R or the Ages and Stages Questionnaire. Thus, we have many different types of neurodevelopmental assessments and thereby cognitive scores reported in our study, which may be difficult to compare and interpret, since the test may assess different cognitive domains and standardisations also vary between countries. An example is the well-debated 8-point difference between the Bayley II and Bayley-III [22]. This has implications for both clinical practice as well as research, where the underestimation of neurodevelopmental disability in regard to the Bayley-III may lead to reduced statistical power in randomised trials. Furthermore, it may vary from site to site whether prematurity is corrected for when conducting the Bayley assessment at 24 months’ corrected age. In extremely preterm infants, scores may differ over 1SD, i.e. up to 20 points, when correcting or not.

Solution: We ask investigators to report if the Bayley score has been corrected for prematurity or not.

For any other assessment than the Bayley-III and Bayley-IV, investigators are asked if the score of the neurodevelopment test is indicative of neurodevelopmental disability. In an ancillary study, standardised mean differences of all assessments used in the SafeBoosC-III follow-up will be compared.

#### Challenge 6: logistics and coordinating

In some sites, the follow-up of infants may be done by neonatal intensive care unit health care professionals whereas in other sites, it may be done in a separate developmental unit with new professionals; thus, the data may not be as easily accessible. Compared to the SafeBoosC-III trial, where all data is collected from the neonatal intensive care unit admission and is typically gathered in one place, the SafeBoosC-III FU relies on more data sources to have a sufficient follow-up percentage. The data collection also relies on more external factors, such as if the parents show up to the consultations and complete the questionnaire. Data may have to be collected across different departments, data systems, hospitals, and more. Furthermore, the SafeBoosC-III study has been going on for several years and may be at risk of ‘study fatigue’ meaning that involved investigators have less motivation to contribute as time goes on.

Solution: The blinded assessor is a health care professional who has not previously been involved in the SafeBoosC-III trial, providing fresh eyes and motivation to the study. The blinded assessor will, typically, be involved in the follow-up of infants, thus creating a connection between the principal investigator in the neonatal intensive care unit and the follow-up clinic where assessments are carried out. We have aimed to minimise extra work in the data collection by developing a simple eCRF which is easy and effective to fill out. Every month we will be sending out newsletters with each site’s data completion rates to promote transparency. Every 6 months, if not otherwise warranted, we will host online steering committee meetings to keep the motivation going.

### A note on parental questionnaires

In this study, we will collect not only clinical data, but also parental-reported data, which may give us valuable information about the child’s development. The sites which do not have routine follow-up clinics can thereby still participate by focusing on parental questionnaires. We utilise both the standardised PARCA-R non-verbal cognitive scale consisting of 34 questions and an additional 11 questions on general health and development, which we can substitute for clinical data if such data are not available, according to the 3-tier model. With this dataset, we will also be able to compare the clinical data with parental-reported data to examine the correlation between what physicians have diagnosed and what the parents remember and have perceived. The health and developmental questionnaire also collects subjective concerns such as if the parents believe their child is thriving. The parental concerns may also reveal any unexpected harms which may not have been observed or voiced in the routine follow-up assessments. Furthermore, we collect information on parental education which is known to be associated with the developmental trajectory of preterm children. We believe that the collection of both clinical data and parental questionnaires will strengthen the final dataset.

We will emphasise the parental questionnaires, as they function as the common denominator in the SafeBoosC-III FU dataset. However, depending on socioeconomic status, motivation, and other external factors, this may be a challenge, and from previous studies, we know that completeness rates of parental questionnaires may be low. Also, we depend on local investigators for reminding, since no contact information is held at the central trial centre. The parents will typically receive a link or QR code directly from the investigator and fill it out online. However, in some sites, Internet access may not be common for parents and therefore it is also possible to introduce the questionnaire in the follow-up clinic and assist the parents in filling it out. It is also possible to call the parents and ask the questions. These solutions will hopefully lead to a higher completeness.

In conclusion, the SafeBoosC-III FU will follow up with trial participants of the SafeBoosC-III trial at 2 years’ corrected age to assess mortality and neurodevelopmental disability following treatment guided by cerebral oximetry during the first 72 h after birth. Most importantly, the SafeBoosC-III FU will ensure an evaluation of any long-term harms. To decrease the risk of generalisation and data-driven biased conclusions, we present a detailed description of the methodology for the SafeBoosC-III follow-up study.

## Trial status

The protocol is registered at clinicaltrials.gov NCT05134116. The first participant was followed up in September 2021 and the anticipated date of study completion is October 2024. Recruitment status can be accessed at www.safeboosc.eu.

### Supplementary Information

**Additional file 1. **

## Data Availability

Not applicable.

## Abbreviations

NIRS: Near-infrared spectroscopy
NDD: Neurodevelopmental disability
NICU: Neonatal intensive care unit
eCRF: Electronic case report form
PARCA-R: Parent Report of Children’s Abilities-Revised

## References

[1] Blencowe H, Cousens S, Chou D, Oestergaard M, Say L, Moller AB (2013). Born too soon: the global epidemiology of 15 million preterm births. Reprod Health.

[2] Blencowe H, Cousens S, Oestergaard MZ, Chou D, Moller A-B, Narwal R (2012). National, regional, and worldwide estimates of preterm birth rates in the year 2010 with time trends since 1990 for selected countries: a systematic analysis and implications. Lancet (British Edition).

[3] Stoll BJ, Hansen NI, Bell EF, Shankaran S, Laptook AR, Walsh MC (2010). Neonatal outcomes of extremely preterm infants from the NICHD Neonatal Research Network. Pediatrics.

[4] Volpe JJ (1997). Brain injury in the premature infant. Neuropathology, clinical aspects, pathogenesis, and prevention. Clin Perinatol..

[5] Vesoulis ZA, Mathur AM (2017). Cerebral Autoregulation, Brain Injury, and the Transitioning Premature Infant. Front Pediatr.

[6] Marret S, Marchand-Martin L, Picaud JC, Hascoet JM, Arnaud C, Roze JC (2013). Brain injury in very preterm children and neurosensory and cognitive disabilities during childhood: the EPIPAGE cohort study. PLoS ONE.

[7] Volpe JJ (2009). Brain injury in premature infants: a complex amalgam of destructive and developmental disturbances. Lancet Neurol.

[8] Saigal S, Morrison K, Schmidt LA (2020). Health, wealth and achievements of former very premature infants in adult life. Semin Fetal Neonatal Med.

[9] Rantakari K, Rinta-Koski OP, Metsaranta M, Hollmen J, Sarkka S, Rahkonen P (2021). Early oxygen levels contribute to brain injury in extremely preterm infants. Pediatr Res.

[10] Hyttel-Sorensen S, Pellicer A, Alderliesten T, Austin T, van Bel F, Benders M (2015). Cerebral near infrared spectroscopy oximetry in extremely preterm infants: phase II randomised clinical trial. BMJ.

[11] Plomgaard AM, Alderliesten T, van Bel F, Benders M, Claris O, Cordeiro M (2019). No neurodevelopmental benefit of cerebral oximetry in the first randomised trial (SafeBoosC II) in preterm infants during the first days of life. Acta Paediatr.

[12] Hansen ML, Pellicer A, Gluud C, Dempsey E, Mintzer J, Hyttel-Sorensen S (2019). Cerebral near-infrared spectroscopy monitoring versus treatment as usual for extremely preterm infants: a protocol for the SafeBoosC randomised clinical phase III trial. Trials.

[13] Pellicer A, Greisen G, Benders M, Claris O, Dempsey E, Fumagalli M (2013). The SafeBoosC phase II randomised clinical trial: a treatment guideline for targeted near-infrared-derived cerebral tissue oxygenation versus standard treatment in extremely preterm infants. Neonatology.

[14] Hansen ML, Pellicer A, Hyttel-Sorensen S, Ergenekon E, Szczapa T, Hagmann C (2023). Cerebral oximetry monitoring in extremely preterm infants. N Engl J Med.

[15] Hansen ML, Pellicer A, Gluud C, Dempsey E, Mintzer J, Hyttel-Sorensen S (2019). Detailed statistical analysis plan for the SafeBoosC III trial: a multinational randomised clinical trial assessing treatment guided by cerebral oxygenation monitoring versus treatment as usual in extremely preterm infants. Trials.

[16] Plomgaard AM, Hagmann C, Alderliesten T, Austin T, van Bel F, Claris O (2016). Brain injury in the international multicenter randomized SafeBoosC phase II feasibility trial: cranial ultrasound and magnetic resonance imaging assessments. Pediatr Res.

[17] Marlow N (2015). Is survival and neurodevelopmental impairment at 2 years of age the gold standard outcome for neonatal studies?. Arch Dis Child Fetal Neonatal Ed.

[18] Chan AW, Tetzlaff JM, Gotzsche PC, Altman DG, Mann H, Berlin JA (2013). SPIRIT 2013 explanation and elaboration: guidance for protocols of clinical trials. BMJ.

[19] Paulson A, Vargus-Adams J (2017). Overview of four functional classification systems commonly used in cerebral palsy. Children (Basel).

[20] Lindsey JC, Brouwers P (1999). Intrapolation and extrapolation of age-equivalent scores for the Bayley II: a comparison of two methods of estimation. Clin Neuropharmacol.

[21] Aylward G, Zhu J. 2019. Available from: https://www.pearsonassessments.com/content/dam/school/global/clinical/us/assets/bayley-4/bayley-4-technical-report.pdf.

[22] Johnson S, Moore T, Marlow N (2014). Using the Bayley-III to assess neurodevelopmental delay: which cut-off should be used?. Pediatr Res.

[23] Blaggan S, Guy A, Boyle EM, Spata E, Manktelow BN, Wolke D (2014). A parent questionnaire for developmental screening in infants born late and moderately preterm. Pediatrics.

[24] Martin AJ, Darlow BA, Salt A, Hague W, Sebastian L, McNeill N (2013). Performance of the Parent Report of Children’s Abilities-Revised (PARCA-R) versus the Bayley Scales of Infant Development III. Arch Dis Child.

[25] Johnson S, Marlow N, Wolke D, Davidson L, Marston L, O'Hare A (2004). Validation of a parent report measure of cognitive development in very preterm infants. Dev Med Child Neurol.

[26] Group ISC (2008). The INIS Study. International Neonatal Immunotherapy Study: non-specific intravenous immunoglobulin therapy for suspected or proven neonatal sepsis: an international, placebo controlled, multicentre randomised trial. BMC Pregnancy Childbirth.

[27] Picotti E, Bechtel N, Latal B, Borradori-Tolsa C, Bickle-Graz M, Grunt S (2020). Performance of the German version of the PARCA-R questionnaire as a developmental screening tool in two-year-old very preterm infants. PLoS One.

[28] Johnson S, Evans TA, Draper ES, Field DJ, Manktelow BN, Marlow N (2015). Neurodevelopmental outcomes following late and moderate prematurity: a population-based cohort study. Arch Dis Child Fetal Neonatal Ed.

[29] Juul SE, Comstock BA, Wadhawan R, Mayock DE, Courtney SE, Robinson T (2020). A randomized trial of erythropoietin for neuroprotection in preterm infants. N Engl J Med.

[30] Natalucci G, Latal B, Koller B, Ruegger C, Sick B, Held L (2016). Effect of early prophylactic high-dose recombinant human erythropoietin in very preterm infants on neurodevelopmental outcome at 2 years: a randomized clinical trial. JAMA.

[31] Broitman E, Ambalavanan N, Higgins RD, Vohr BR, Das A, Bhaskar B (2007). Clinical data predict neurodevelopmental outcome better than head ultrasound in extremely low birth weight infants. J Pediatr.

[32] O'Shea TM, Kuban KC, Allred EN, Paneth N, Pagano M, Dammann O (2008). Neonatal cranial ultrasound lesions and developmental delays at 2 years of age among extremely low gestational age children. Pediatrics.

[33] Cainelli E, Arrigoni F, Vedovelli L (2020). White matter injury and neurodevelopmental disabilities: a cross-disease (dis)connection. Prog Neurobiol.

[34] Panfoli I, Candiano G, Malova M, De Angelis L, Cardiello V, Buonocore G (2018). Oxidative stress as a primary risk factor for brain damage in preterm newborns. Front Pediatr.

[35] Weinberger B, Laskin DL, Heck DE, Laskin JD (2002). Oxygen toxicity in premature infants. Toxicol Appl Pharmacol.

[36] Marlow N, Doyle LW, Anderson P, Johnson S, Bhatt-Mehta V, Natalucci G (2019). Assessment of long-term neurodevelopmental outcome following trials of medicinal products in newborn infants. Pediatr Res.

[37] Howe CJ, Cole SR, Lau B, Napravnik S, Eron JJ (2016). Selection bias due to loss to follow up in cohort studies. Epidemiology.

[38] Piedvache A, van Buuren S, Barros H, Ribeiro AI, Draper E, Zeitlin J (2021). Strategies for assessing the impact of loss to follow-up on estimates of neurodevelopmental impairment in a very preterm cohort at 2 years of age. BMC Med Res Methodol.

